# Case Report: Xsens motion analysis and virtual reality–based rehabilitation in a national-level badminton player following MPFL reinjury leading to postoperative patellar fracture

**DOI:** 10.3389/fspor.2025.1667384

**Published:** 2026-01-02

**Authors:** Chaitali S. Vikhe, Nikita Bhusari, Swapnil U. Ramteke

**Affiliations:** 1 Department of Sports Physiotherapy, Ravi Nair Physiotherapy College, Datta Meghe Institute of Higher Education and Research (Deemed to be University), Wardha, Maharashtra, India; 2 Department of Musculoskeletal Physiotherapy, Ravi Nair Physiotherapy College, Datta Meghe Institute of Higher Education and Research (Deemed to be University), Wardha, Maharashtra, India

**Keywords:** recurrent patellar dislocation, virtual reality rehabilitation, gait analysis, Xsens motion analysis, sports physiotherapy

## Abstract

**Introduction:**

Recurrent patellar dislocation presents a significant rehabilitation challenge, particularly in athletes engaged in high-demand, multi-directional sports such as badminton. This case report describes a complex presentation in a state-level athlete with multiple patellar dislocations, followed by medial patellofemoral ligament (MPFL) reconstruction and a subsequent comminuted patellar fracture. The case was further complicated by proprioceptive deficits, extension lag, sensory changes, and kinesiophobia, all of which limited the athlete's functional performance and return to sport.

**Case presentation:**

A 23-year-old male National-level badminton player reported persistent functional limitations after recurrent patellar dislocations and two surgeries (MPFL reconstruction and patellar fracture fixation using K-wires). Clinical examination revealed quadriceps atrophy, patellar hypomobility, altered infrapatellar sensation, genu valgum, proprioceptive errors, and fear of dynamic movements. Functional impairments included difficulty with squatting, running, and agility. Gait analysis using the Xsens system confirmed altered joint kinematics and asymmetrical movement patterns.

**Intervention:**

A comprehensive, phase-wise rehabilitation program was designed targeting biomechanical, neuromuscular, and psychological recovery. Key components included quadriceps reactivation, proprioceptive retraining, neuromuscular electrical stimulation (*N*MES), agility drills, sensory re-education, and mindfulness-based training. Virtual reality (VR)-based rehabilitation using *Oculus Quest* (RehabVR) was introduced to enhance neuro-motor integration and patient engagement. Objective readiness was tracked via functional testing and motion analysis.

**Outcomes:**

The athlete showed marked recovery in pain-free range of motion (0°–125°), quadriceps strength (MMT Grade 5), and proprioceptive precision (joint position error reduced from 6.3° to 2.1°). KOOS improved from 62 to 100, and LEFS from 55 to 80. Xsens gait analysis demonstrated improved step length symmetry, gait velocity (1.32 m/s), normalized foot progression, and restored center of mass balance. Kinesiophobia resolved, and the athlete returned safely to non-contact sport activities.

**Conclusion:**

This case emphasizes the need for individualized, multidisciplinary rehabilitation in athletes with recurrent patellar instability and surgical complications. Integration of virtual reality, motion analysis, and mindfulness training supported neuromuscular recovery and psychological readiness.

## 1 Introduction

Patellar instability is a common clinical concern in young, physically active populations, particularly athletes engaged in sports requiring rapid changes of direction, jumping, and deceleration, such as badminton, basketball, and soccer. Lateral patellar dislocation is the most frequent type, accounting for nearly 90% of cases, and often recurs if not managed appropriately (1–3). The incidence of first-time dislocation ranges from 5.8 to 77 per 100,000 persons per year, with adolescents and young athletes being most affected. Recurrence rates can be as high as 39%, particularly in individuals with anatomical or biomechanical risk factors (4, 5).

The medial patellofemoral ligament (MPFL) is the primary static stabilizer preventing lateral patellar displacement during the initial 30° of knee flexion. Injury to the MPFL occurs in nearly all first-time lateral dislocations, making it critical to assess and, when necessary, reconstruct during surgical intervention (6–8). Recurrent dislocations compromise patellofemoral congruency, damage articular cartilage, and predispose athletes to long-term complications such as early-onset osteoarthritis (9, 10). Predisposing factors include trochlear dysplasia, patella alta, increased tibial tubercle–trochlear groove distance, ligamentous laxity, and an elevated Q-angle, which increases lateral forces on the patella (11, 12).

High-demand sports like badminton impose significant biomechanical stress on the patellofemoral joint through high-speed lunges, multidirectional agility, eccentric loading, and single-leg landings (13–15). These activities require precise patellar tracking, neuromuscular control, and proprioception for both performance and injury prevention. Persistent instability can impair force generation, compromise alignment during rapid deceleration, and elevate the risk of re-injury, chronic pain, and kinesiophobia (16, 17).

This case is rare and clinically significant due to the combination of multiple factors: the patient is a national-level badminton athlete, experienced recurrent lateral patellar dislocations despite prior surgical intervention, sustained a subsequent traumatic patellar fracture, and had delayed or interrupted rehabilitation. These combined factors posed unique challenges for restoring knee function, proprioception, and return-to-sport capacity, distinguishing this case from typical patellar instability presentations.

The report details a phased, functionally graded, individualized rehabilitation protocol integrating pain management, neuromuscular retraining, plyometric control, proprioceptive re-education, and psychological readiness (18, 19). Clinical decisions were guided by objective outcome measures, including manual muscle testing, joint position sense, hop tests, the Balance Error Scoring System, agility assessments, and advanced biomechanical analysis using the Xsens motion capture system. These assessments provided quantitative data to guide progression, identify asymmetries, and validate readiness for high-demand athletic activity, emphasizing that optimal recovery in high-performance athletes involves not only tissue healing but also restoring confidence, coordination, and competitive readiness.

### 1.1 Case presentation

A 23-year-old male national-level badminton player presented to the Sports Physiotherapy Outpatient Department with persistent functional limitations following recurrent patellar dislocations and two surgical interventions. The patient provided informed consent for the use of clinical and therapeutic data for academic and publication purposes. He had no major comorbidities and had previously competed at the national level without limitations.

The initial injury occurred in April 2023 during a jump smash, leading to a spontaneous lateral patellar dislocation. Two recurrent dislocations followed in July 2023 and April 2024. MRI revealed a grade II medial patellofemoral ligament (MPFL) tear, and primary MPFL reconstruction using a semitendinosus graft was performed in May 2024. In July 2024, the patient sustained a patellar fracture during rehabilitation, necessitating a second surgery in August 2024 involving K-wire fixation and MPFL revision.

#### 1.1.1 Post-surgical rehabilitation history

The patient underwent a standard early postoperative physiotherapy program from August to November 2024 (approximately 10–12 weeks). Early rehabilitation focused on edema control, pain management, gradual ROM recovery, and quadriceps activation. However, adherence fluctuated due to academic commitments and relocation, and he discontinued therapy prematurely in late November 2024 before reaching functional strengthening and sports retraining phases. Because rehabilitation stopped before advanced stages, the patient did not attempt a structured return-to-sport (RTS) and instead self-limited activity to basic ADLs.

Between December 2024 and April 2025, the athlete engaged only in intermittent home exercises without supervision. He reported progressive stiffness, difficulty with single-leg loading, avoidance of dynamic drills, and increasing apprehension during cutting or landing tasks. Persistent limitations affected university sports participation, prompting his orthopedic surgeon and team physiotherapist to refer him back for comprehensive reassessment and rehabilitation in May 2025.

#### 1.1.2 Status at Re-presentation (Pre-intervention assessment)

On returning in May 2025, the patient demonstrated significant functional impairments, including Genu valgum and inability to squat, reduced running tolerance, inability to sit cross-legged, incomplete knee extension and flexion, altered infrapatellar sensation, and fear of dynamic movements. These deficits are summarized in Table 1, which details pre-intervention ROM, muscle strength, functional capacity, and kinesiophobia scores.

**Table 1:** Clinical outcome measures pre- and post-rehabilitation.

Domain	Assessment Tool/Method	Pre-Rehabilitation Findings	Post-Rehabilitation Findings	Interpretation
Pain	Numeric Pain Rating Scale (NPRS)	7/10 during the activity	0/10 during activity	Significant reduction in pain intensity
Knee ROM (Active)	Goniometer	0°–90°	0°–125°	Improved flexion range, nearing functional range
Extension Lag	Observation in a high sitting	10°	2°	Almost resolved; quadriceps strength improved
Quadriceps Strength	Manual Muscle Testing (MMT)	Grade 3+	Grade 5	Improved strength
Hip Abductor Strength	MMT	Grade 4	Grade 5	Full recovery in hip stability support
Patellar Mobility	Medial/lateral/superior glide palpation	Hypomobile (superior restriction)	Normalized mobility	Reduced risk of anterior knee pain recurrence
Joint Position Sense (JPS)	Goniometric repositioning task (30° angle)	6.3° error	2.1° error	Marked improvement in proprioceptive accuracy
Scar Sensitivity	Light touch, pressure palpation	Tender, hypersensitive over medial incision	None	Desensitization achieved
Kinesiophobia	Clinical interview (subjective)	Moderate fear of reinjury	None	Improved confidence in movement
Sensory Deficit	Monofilament, proprioception (manual)	Reduced light touch, altered proprioception over the peripatellar area	Intact	Suggests nerve recovery or cortical reorganization
Agility/Hop Test	Single-leg hop for distance	Unable to perform	Able to perform without compensation	Return of neuromuscular control and impact tolerance

A detailed biomechanical evaluation and 3D gait analysis using the Xsens Motion Capture System were performed pre- and post-rehabilitation (Table 2). Pre-intervention findings included decreased stance phase duration, reduced peak knee flexion during swing, excessive foot external rotation, reduced propulsion symmetry, and altered COM acceleration patterns.

**Table 2:** Xsens motion analysis findings—pre- and post-rehabilitation.

Gait Parameter	Pre-Rehabilitation	Post-Rehabilitation	Normal Reference Range	Interpretation
Gait Speed	0.96 m/s	1.32 m/s	1.2–1.4 m/s	Improved to normal; reflects restored confidence and propulsion
Cadence	114.8 steps/min	126.3 steps/min	120–130 steps/min	Normal cadence regained, indicative of symmetrical limb movement
Step Length	L: 51.2 cm; R: 50.5 cm	L: 67.4 cm; R: 66.8 cm	65–75 cm	Step symmetry and stride length normalized
Stance Phase	L: 39.5%; R: 43.6%	L: 59.3%; R: 60.1%	∼60%	Balanced stance time, offloading corrected
Foot Strike Pattern	Toe strike bilaterally	Heel-to-toe	Heel-to-toe	Normalized strike pattern achieved
Peak Knee Flexion (Swing)	L: 69.7°; R: 66.4°	L: 78.2°; R: 76.9°	75°–85°	Improved knee mobility during the swing phase
Knee Rotation	Excessive external rotation bilaterally	Mild ER within normal functional range	Neutral to mild ER	Compensation reduced; proprioception and control enhanced
Pelvic Obliquity	Elevated on the left side	Within 4° symmetry	<5°	Pelvic control restored
Foot Progression Angle	L: –50.3°; R: –32.7°	L: –13.2°; R: –11.6°	∼10°–15° outward	Improved foot alignment reflects enhanced neuromuscular control
COM Acceleration	Reduced on the left side	Symmetric anterior-posterior shift	Symmetrical, stable	Restored balance and propulsion

**Figure 1:**
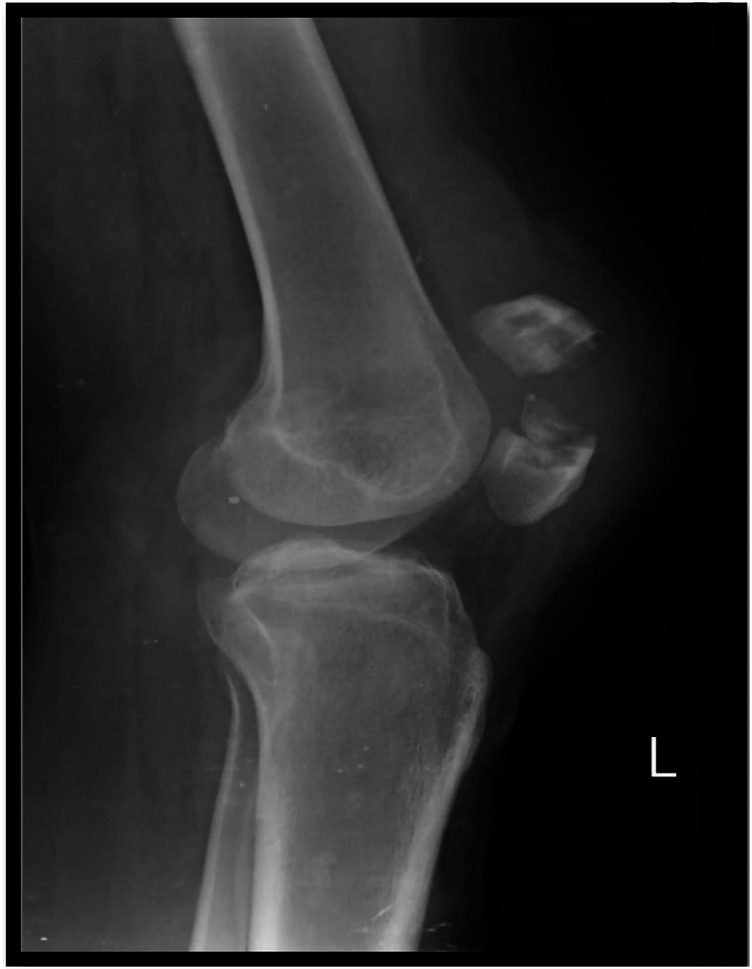
Shows the lateral x-ray of the left knee demonstrates a displaced transverse fracture of the patella, located at the mid-patellar level and there is displacement of fracture fragments, with the superior and inferior poles no longer in anatomical alignment.

**Figure 2:**
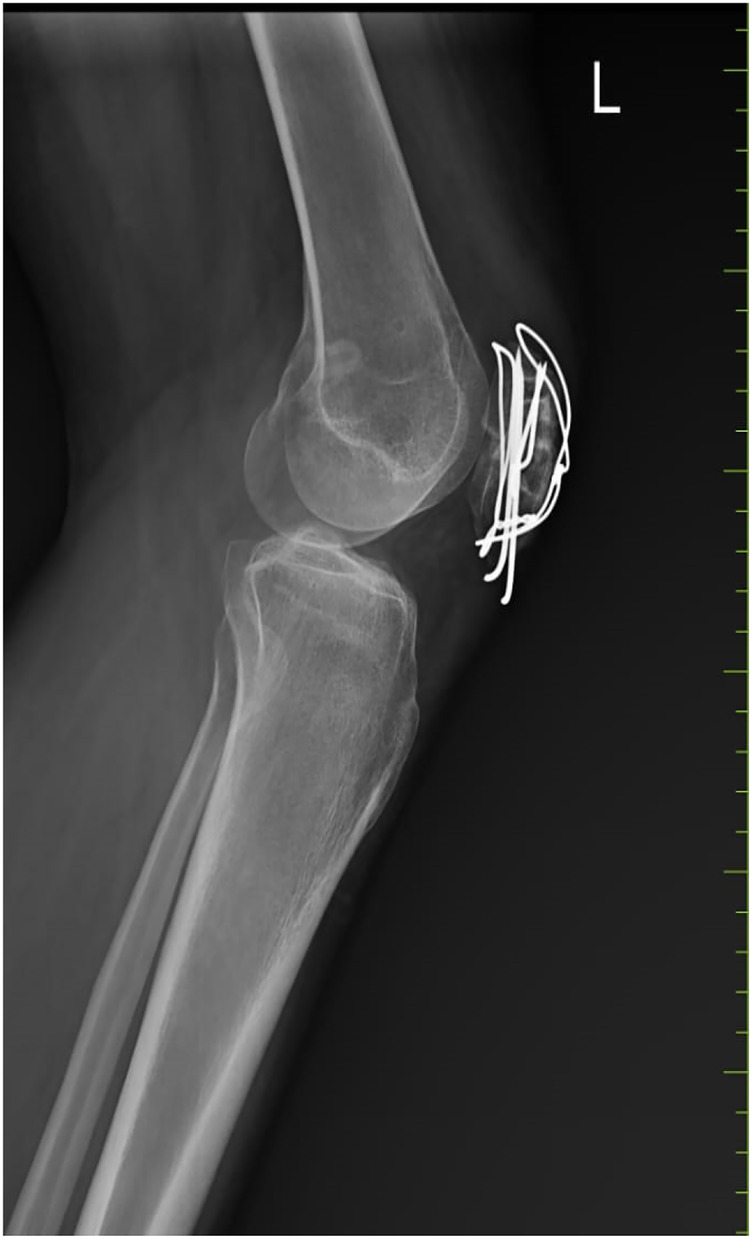
Shows the radiographs reveal a previously displaced transverse fracture of the patella that has been surgically stabilized.

**Figure 3:**
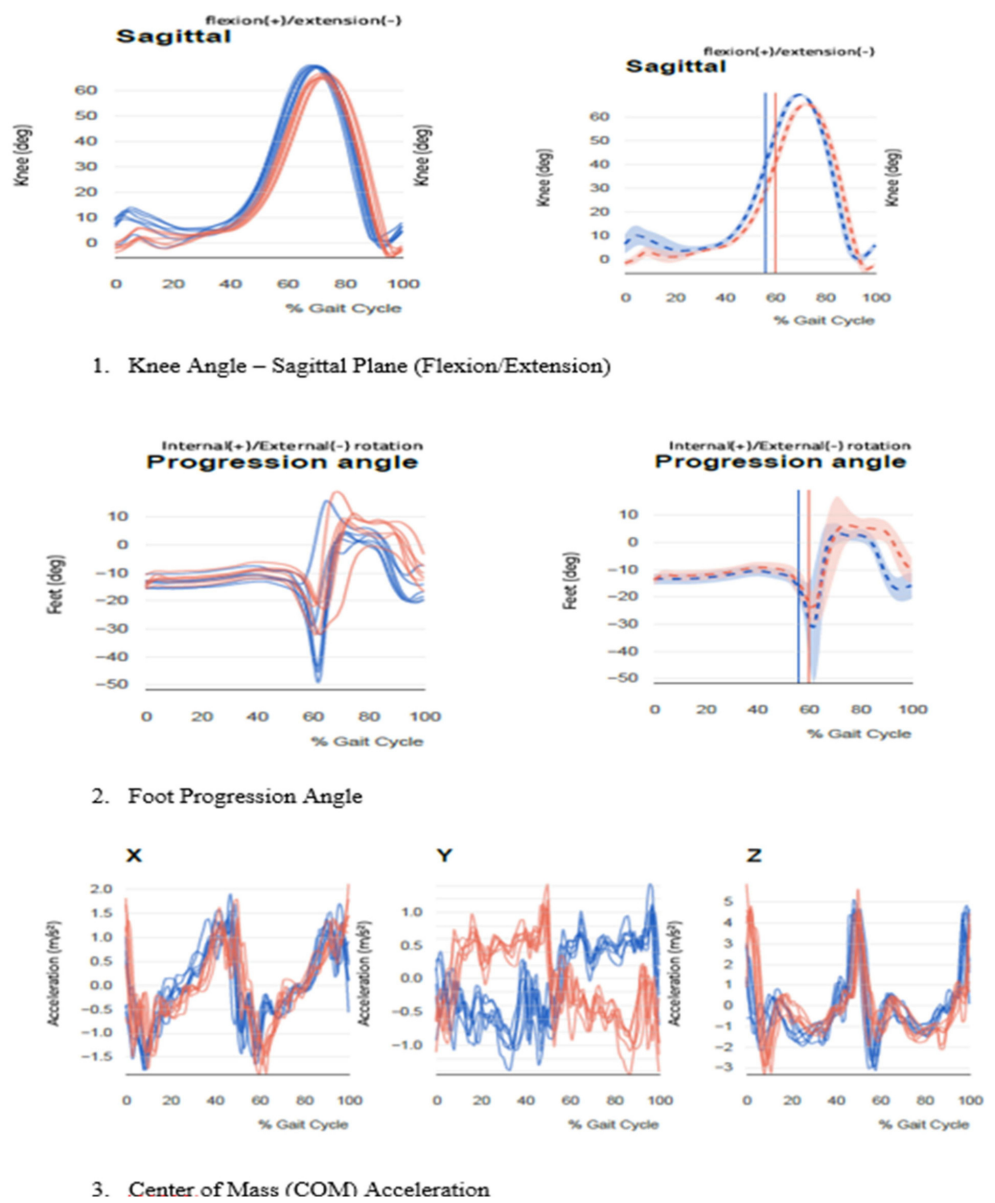
Gait analysis using Xsens motion capture pre- and post-rehabilitation. (1) Knee joint angle in the sagittal plane shows increased peak flexion during swing and improved terminal extension at heel strike, indicating recovery from extension lag and enhanced functional mobility. (2) Foot progression angle reveals a significant reduction in excessive external rotation post-rehabilitation, reflecting improved lower limb alignment and neuromuscular control. (3) Center of Mass (COM) acceleration in the vertical **(Z)** direction demonstrates restored propulsion symmetry and improved gait stability.

Figures 1, 2 display pre- and post-operative radiographs. Figure 3 illustrates pre- and post-rehabilitation Xsens gait changes, demonstrating:

1. Improved sagittal knee kinematics, including recovery from terminal extension lag and greater peak flexion in swing.
2. Reduced excessive external foot progression angle, indicating improved limb alignment and neuromuscular control.
3. Restored COM vertical acceleration symmetry, reflecting improved gait stability and propulsion.

### 1.2 Intervention

A multimodal, phase-wise rehabilitation protocol was administered over 12 weeks, focusing on neuromuscular control, functional mobility, proprioceptive retraining, psychological readiness, and sports-specific reconditioning.

A head-mounted Oculus Quest VR system was used to deliver immersive rehabilitation tasks. The selected VR applications provided interactive, gamified movement training and simulated sport-specific environments. Tasks included:

- Visual feedback balance and coordination exercises (dynamic joint repositioning, obstacle avoidance).
- Sports-specific lower limb movements (single-leg stance control, multidirectional lunges, virtual agility drills).
- Cognitive-motor dual tasks to enhance neuromuscular coordination under mental load.
- Mindfulness modules incorporating guided breathing and grounding tasks in immersive VR landscapes to reduce kinesiophobia and promote body awareness.

Session Frequency: 3 times per week.

Duration: 20–25 min per session.

Progression Criteria: Ability to perform dynamic drills confidently with accurate proprioceptive control and minimal compensations. Table 3 shows Structured Rehabilitation Interventions for Post-MPFL Reconstruction and Patellar Fracture.

**Table 3:** Structured rehabilitation interventions for post-MPFL reconstruction and patellar fracture.

Goal	Intervention	Rationale	Dosage & Frequency	Progression Criteria
Pain Management	Cryokinetics, TENS, Patellar taping, Soft tissue release	Alleviate discomfort, facilitate active participation	TENS: 10 min; Taping: 8 h max; Cryo: post-exercise	Pain 0/10
Quadriceps Strength	TKE, Multi-angle SLR, Eccentric step downs, NMES	Restore extensor strength and patellar alignment	3 × 10–15 reps; NMES 3x/week	Near-normal strength, no lag
ROM Restoration	Wall slides, Stationary cycling, MWM, Stretching	Achieve full flexion/extension without pain	ROM to 125° flexion within 6 weeks	Lag ≤2°, Flexion ≥125°
Core & Hip Control	Monster walks, Bridges, Side planks, Dead bug	Reduce dynamic valgus and trunk sway	3 sets of 10–15, 3x/week	Maintained during single-leg tasks
VR-Enhanced Proprioception	Visual feedback balance tasks, Targeted movement control (via VR)	Increase accuracy in JPS, rebuild movement confidence	20 min, 3x/week	JPS error ≤2.5°
Sensory Re-education	Texture desensitization, Tapping, Sensory discrimination tasks	Improve altered sensation and cutaneous awareness	2x/day, 5–10 min	Return of intact sensation
Mindfulness Training	Breath focus, Body scan, Movement meditation	Reduce fear, enhance body awareness	15 min/day, 5 days/week	Psychological readiness for loading
Functional Re-integration	Sit-to-stand, Walk-jog drills, Dynamic squats, Sports drills in VR	Reintroduce real-world activities and dynamic loads	Progressive, 10–30 min/day	Able to perform jog → sport-specific drills
Plyometric Agility	Double/single-leg hops, Crossover hops	Assess neuromuscular control and readiness to return	3 × 5 reps, 3x/week	LSI ≥90%, no compensatory movement

## 2 Results

Following the implementation of a comprehensive rehabilitation protocol that included conventional physiotherapy, proprioceptive neuromuscular training, and immersive virtual reality (VR)-based therapy using *Oculus Quest*, the patient demonstrated substantial improvements across clinical, functional, psychological, and biomechanical parameters. As shown in Table 1, pain intensity during activities decreased from 7/10 to 0/10 on the Numeric Pain Rating Scale (NPRS), indicating complete pain resolution. Knee active range of motion improved from a restricted 0°–90° to a functional 0°–125°, and the observed extension lag reduced from 10° to just 2°, reflecting significant gains in quadriceps control and joint mobility.

Manual Muscle Testing (MMT) revealed strength recovery in both the quadriceps and hip abductors, with grades improving from 3+ and 4 to full strength (Grade 5), respectively. Proprioceptive accuracy, as measured through joint position sense (JPS), showed marked enhancement with a decrease in error from 6.3° to 2.1°. Patellar mobility normalized from an initially hypomobile superior glide restriction, reducing the risk of anterior knee stress recurrence. The patient also achieved full desensitization over previously hypersensitive surgical scars, along with restored light touch and proprioception around the peripatellar area, suggestive of peripheral nerve recovery or cortical reorganization. Notably, psychological improvements were observed, including the complete resolution of moderate kinesiophobia and a return of movement confidence.

**Figure 4:**
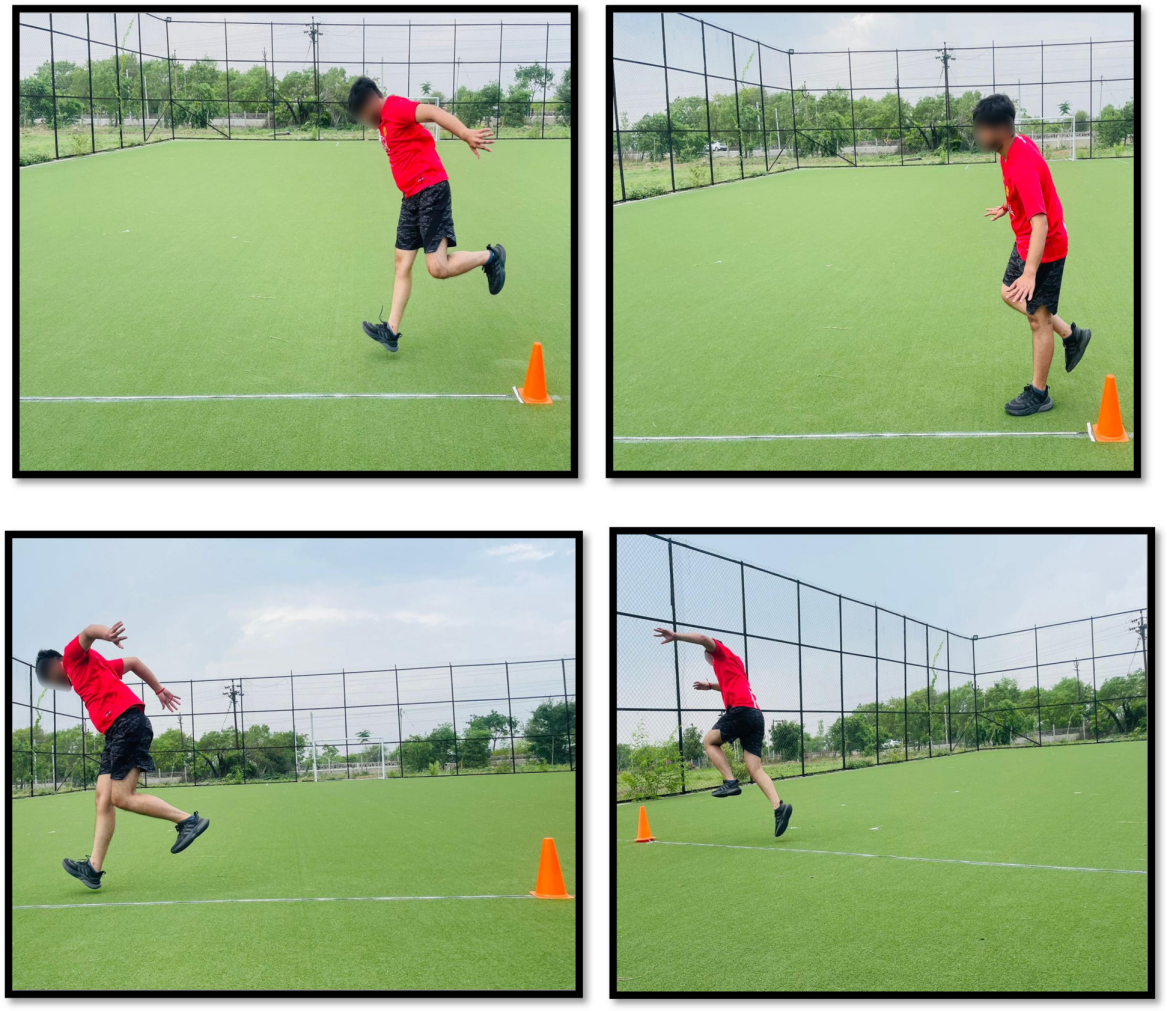
Assessment of hop test performance before rehabilitation.

Functionally, the patient progressed from being unable to perform a single-leg hop shown in Figure 4 to executing it without compensatory patterns, indicating restored neuromuscular control and impact tolerance. These findings are summarized in Table 1.

Three-dimensional motion analysis conducted with Xsens technology. As illustrated in Figure 3, post-rehabilitation gait analysis revealed significant improvements in key biomechanical parameters. The knee sagittal angle (Figure 3.1) displayed a marked increase in peak flexion during the swing phase (from 66.4° to 76.9° on the right), and restoration of terminal extension at heel strike, reflecting enhanced quadriceps strength and reduced extension lag. The foot progression angle (Figure 3.2) improved notably, with external rotation reducing from −50.3° to −13.2° on the left side, indicating better limb alignment and dynamic control. Additionally, the COM vertical acceleration (Figure 3.3) exhibited more symmetrical and stable patterns across the gait cycle, suggesting restored balance and propulsion mechanics.

Further confirmed the biomechanical improvements achieved post-rehabilitation (Table 2). Gait speed increased from 0.96 m/s to 1.32 m/s, aligning with the normative range of 1.2–1.4 m/s. Cadence improved from 114.8 to 126.3 steps/min, and step length increased bilaterally (L: 51.2 cm–67.4 cm; R: 50.5 cm–66.8 cm), indicating improved stride symmetry and propulsion mechanics. Stance phase distribution, previously asymmetrical (L: 39.5%; R: 43.6%), was normalized to 59.3% and 60.1%, respectively. The patient's foot strike pattern shifted from a compensatory toe strike bilaterally to a heel-to-toe pattern, and peak knee flexion during the swing phase improved bilaterally, approaching the normative range of 75°–85°. Abnormal external knee rotation, elevated pelvic obliquity, and altered foot progression angles (L: −50.3°; R: −32.7°) all normalized post-intervention, indicating enhanced limb alignment and motor control. Center of mass acceleration, previously reduced on the left side, became symmetrical, reflecting improved balance and coordinated propulsion.

The integration of immersive VR training via Oculus Quest further supported neuromuscular re-education and psychological engagement. Virtual rehabilitation tasks simulated dynamic sporting scenarios, enabling safe, graded exposure to movement, which contributed to improved proprioceptive feedback, sensory integration, and movement confidence. The patient showed increased responsiveness during virtual body-awareness tasks and improved agility and reaction time during virtual sports drills, suggesting the effectiveness of VR in bridging the gap between clinic-based therapy and sport-specific demands.

Patient-reported outcome measures also reflected significant recovery. The KOOS score increased from 62 to 100, indicating complete functional normalization across pain, symptoms, activities of daily living, and sport/recreation subscales. The Lower Extremity Functional Scale (LEFS) improved from 55 to 80, reflecting near-complete restoration of lower limb function. Moreover, the Tampa Scale for Kinesiophobia (TSK) decreased from 68 to 30, denoting a reduction in fear-avoidant behaviors and enhanced psychological readiness for return to sport.

Taken together, these outcomes—supported by data from Tables 1, 2—highlight the effectiveness of a comprehensive, individualized rehabilitation program incorporating conventional physiotherapy, technology-assisted VR, proprioceptive training, and psychological support in facilitating successful recovery and return to sport following MPFL reconstruction and patellar fracture fixation.

## 3 Discussion

Recurrent patellar dislocation is a debilitating condition, particularly among young athletes engaged in high-impact, multi-directional sports such as badminton, which demands rapid acceleration, deceleration, sudden directional changes, jumping, and landing (20, 21). These complex movement patterns require optimal joint alignment, proprioceptive acuity, and neuromuscular coordination. Any instability in the patellofemoral joint compromises the kinetic chain, leading to compensatory mechanisms and increasing the risk of further injuries or performance deficits.

This case is clinically significant because it illustrates the compounded challenges following recurrent patellar dislocations, MPFL insufficiency, and subsequent complications, including a patellar fracture. Such injuries result in structural deficits (e.g., patellar maltracking, reduced mobility), neuromuscular dysfunction (e.g., quadriceps weakness, extension lag), proprioceptive disturbances, and psychological barriers such as kinesiophobia. Consequently, the athlete's confidence, agility, and functional performance were markedly reduced.

Traditional rehabilitation methods for recurrent patellar instability generally focus on quadriceps strengthening, VMO activation, proprioceptive exercises, and gradual return-to-sport drills. Early mobilization and progressive resistance training are emphasized to prevent stiffness, quadriceps inhibition, and maltracking (22–26). Studies by Nomura et al. and Sappey-Marinier et al. highlight the importance of timely rehabilitation, VMO activation, and patellar alignment exercises to support ligament healing (27, 28). Standard functional criteria, such as limb symmetry index (LSI) > 90% and functional testing (Hop tests, *T*-Test, BESS), are commonly used to guide safe return to sport (29).

However, in this case, post-operative immobilization due to a patellar fracture, along with delayed or interrupted rehabilitation, led to pronounced quadriceps atrophy, proprioceptive deficits, and functional decline. These challenges deviated from standard recovery trajectories and required a more nuanced, multidisciplinary approach.

### 3.1 Enhanced rehabilitation strategy in this case

A phased, individualized protocol addressed both biomechanical and biopsychosocial factors. Interventions included VMO activation, neuromuscular electrical stimulation, proprioceptive retraining, agility drills, mindfulness training, and functional testing. Early identification of sensory deficits and asymmetries enabled tailored intervention planning. Immersive Virtual Reality (VR) using Oculus Quest was integrated in later phases to simulate high-speed, dynamic sports environments, improve motor planning, enhance sensory integration, and reduce kinesiophobia. This innovative approach complemented traditional methods, providing engagement, neuroplasticity benefits, and confidence-building, which are often not addressed in conventional rehabilitation.

Gait analysis using Xsens confirmed biomechanical recovery, with improvements in gait speed, cadence, stride length, foot progression angles, and center-of-mass acceleration. These improvements reflected restored neuromuscular symmetry, motor relearning, and readiness for return to play.

Psychological and neurological components were systematically addressed through mindfulness, functional exposure, and sensory re-education. These interventions were critical in overcoming fear of re-injury, altered infrapatellar sensation, and proprioceptive errors—factors often underreported in patellofemoral injury literature but pivotal in this case.

Key challenges included:

- Delayed rehabilitation post-MPFL reconstruction, causing missed conditioning opportunities.
- Surgical re-intervention and immobilization leading to quadriceps inhibition and scar formation.
- Psychological barriers limiting early progression.
- Sensory impairments affecting postural control and agility.
- Need for cautious, individualized return-to-sport criteria due to layered impairments.

Key lessons and clinical implications:

1. Rehabilitation must be athlete-specific, goal-oriented, and incorporate psychosocial components.
2. Delayed or incomplete rehabilitation increases risk of functional decline and re-injury.
3. Functional and sensory testing should be embedded into clinical pathways for decision-making.
4. Interdisciplinary collaboration among orthopedic surgeons, physiotherapists, and sports psychologists enhances outcomes.
5. Technology-based tools, such as VR and motion analysis systems (e.g., Xsens), provide objective, engaging platforms to complement traditional rehabilitation and facilitate neuro-muscular recovery.

### 3.2 Limitations and contingency

This case report has several inherent limitations. First, as a single-case study, the findings may not be generalizable to all athletes or patients with recurrent patellar dislocation and post-surgical complications. Second, the absence of a control or comparison group limits the ability to definitively attribute improvements solely to the VR-assisted or multidisciplinary interventions. Third, some assessments, such as psychological readiness and proprioception, may be influenced by subjective factors, despite using validated scales.

Contingency planning was critical throughout rehabilitation. Potential setbacks—including delayed healing, post-surgical complications, quadriceps inhibition, and kinesiophobia—were mitigated through phased, individualized interventions, frequent monitoring of functional and biomechanical parameters, and adjustments to exercise intensity. Close collaboration with orthopedic surgeons, physiotherapists, and sports psychologists allowed for rapid response to emerging issues, ensuring safe progression and minimizing the risk of re-injury.

Overall, while this case provides valuable insights into complex knee rehabilitation, caution is advised in extrapolating these findings beyond similar clinical scenarios, and further studies with larger cohorts are warranted to validate these outcomes.

## 4 Conclusion

This case highlights the clinical complexity of recurrent patellar dislocation complicated by MPFL insufficiency, reconstruction, and traumatic patellar fracture in a competitive athlete. Individualized, multidisciplinary rehabilitation including strength training, proprioceptive retraining, mindfulness, and VR-assisted interventions led to meaningful improvements in pain, range of motion, muscle strength, proprioception, psychological readiness, and functional performance. Objective measures (KOOS, LEFS, TSK, JPS error, gait metrics) confirmed recovery and guided safe return-to-play. The case underscores the value of evidence-based, technology-enhanced rehabilitation and the importance of timely, goal-directed interventions for successful reintegration into high-performance sport.

## Data Availability

The datasets generated and analyzed during the current study (including clinical outcome measures and motion analysis data) are available from the corresponding author on reasonable request.
